# The RNA revolution in medicine: from gene regulation to clinical therapeutics

**DOI:** 10.1080/19768354.2025.2548253

**Published:** 2025-08-25

**Authors:** Jiwon Jeong, Sunjoo Jeong

**Affiliations:** Laboratory of RNA Cell Biology, Department of Bioconvergence Engineering, Dankook University, Yongin, Republic of Korea

**Keywords:** RNA, antisense oligonucleotides, splicing switching oligonucleotides, RNA interference, aptamers

## Abstract

Breakthrough discoveries in RNA biology have led to a paradigm shift in our understanding of RNA—from passive intermediates to active regulators of gene expression. Technological innovations and deeper insights into RNA regulation have transformed the field, positioning RNA as a powerful tool for therapeutic development. Recent advances in RNA technologies have revolutionized medicine by enabling the precise targeting of specific mRNAs to modulate aberrant transcripts, correct genetic defects, and reprogram cellular behavior. This review provides an overview of the coordinated regulation of mRNA processing and its application to RNA-based therapeutics, including antisense oligonucleotides (ASOs), splice-switching oligonucleotides (SSOs), small interfering RNAs (siRNAs), and Aptamers. We focus on clinically approved RNA therapeutics, emphasizing their biological mechanisms such as RNA stability and splicing regulation. The expanding repertoire of RNA technologies underscores the translational potential of RNA biology and its growing clinical impact. Future developments are expected to yield highly specific, modular, and programmable RNA medicines capable of treating a wide range of previously intractable diseases.

## 1. Introduction

Over the last few decades, RNA has emerged from being a passive messenger of genetic information to a dynamic regulator of cellular processes and a powerful therapeutic modality. This transformation began with foundational discoveries in the mid-twentieth century, including the identification of RNA as a single-stranded molecule by Rich and Davies in 1956 and the discovery of messenger RNA (mRNA) by Jacob and Monod in 1961. Over time, RNA molecules have been recognized not only as passive participants in processes such as transcription, splicing, and translation but also as key players in post-transcriptional regulation and cellular signaling. Landmark advances, such as the discovery of RNase H-mediated RNA cleavage in 1969, RNA splicing in 1977, microRNAs (miRNAs) in 1993, the mechanism of RNA interference (RNAi) in 1998, and CRISPR-based RNA editing in 2016, underscored the functional versatility of RNA and opened new avenues for therapeutic development.

**Figure 1.**
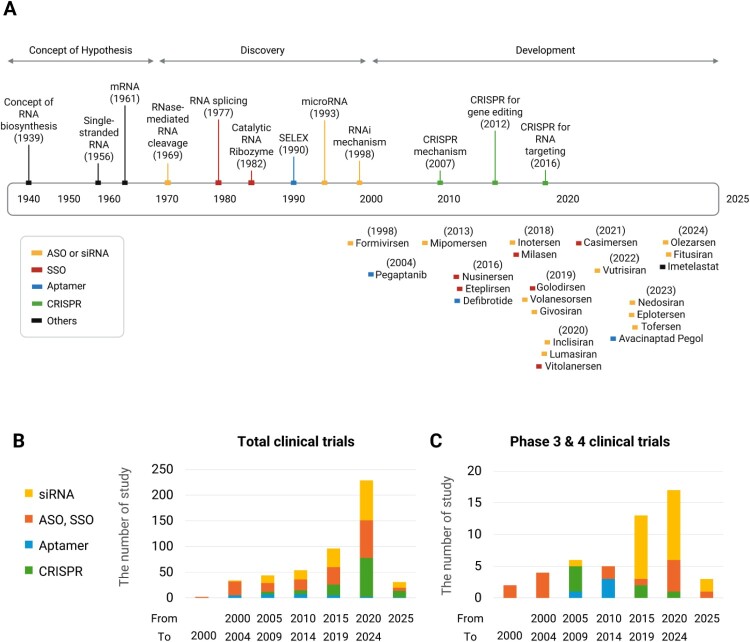
An overview of RNA biology and RNA-based therapeutics. (A) Timeline of RNA-based therapeutic development and key discoveries. This timeline illustrates the progression of RNA-based therapeutics from the initial concept and hypothesis stage through discovery and development, from the 1940s to 2025. The timeline is divided into three main phases: concept of hypothesis, discovery, and development. (B) Number of clinical trials initiated for RNA-based therapeutics categorized by modality: antisense oligonucleotides (ASOs, yellow) and small interfering RNA (siRNA, yellow), splicing-switching ASOs (SSOs, red), aptamers (blue), clustered regularly interspaced short palindromic repeats (CRISPR, green), and others (black). Data were obtained from ClinicalTrials.gov. (C) Number of later-stage clinical trials (Phases III and IV) across the same RNA modalities. Data were obtained from ClinicalTrials.gov.

The timeline of RNA therapeutics—from the initial identification of RNA in the 1950s to the development of sophisticated gene-modulating drugs in the 2020s—illustrates this extraordinary scientific and clinical evolution (Figure 1A). The development phase of RNA therapeutics began with the approval of fomivirsen in 1998, the first FDA-approved RNA-based drug, used to treat cytomegalovirus (CMV) retinitis in immunocompromised patients. This milestone marked a shift for RNA, from a subject of basic molecular biology research to a clinically actionable modality. In the years that followed, clinical trials notably led to the approval of the RNA aptamer pegaptanib in 2004 for age-related macular degeneration (AMD).

As shown in Figure 1B and 1C, the clinical development of RNA therapeutics has rapidly expanded over the last two decades. This growth reflects the increasing maturity of RNA modalities in drug design, delivery, and safety. Since then, antisense oligonucleotides (ASOs), small interfering RNAs (siRNAs), and aptamers have progressed from the bench to the bedside, culminating in the approval of numerous RNA-based drugs, including nusinersen, eteplirsen, and fitusiran (Quemener et al. (2020)). Furthermore, the global deployment of mRNA vaccines to combat SARS-CoV-2 has demonstrated safety and efficacy.

This review provides an overview of RNA-based therapeutics, emphasizing their biological and recent technological progress. We focus on clinically approved RNA technologies, such as ASOs, RNA interference, and RNA aptamers (Table 1). We discuss how fundamental research in RNA biology has guided therapeutic development and review FDA-approved RNA-based drugs that are transforming modern medicine.

**Table 1.** FDA-approved RNA therapeutics.

Type	Mechanism of action	Generic name	Brand name	Company	FDA approval year	Sequence (5’ to 3’)	Indication	Target gene/protein	Delivery
		Patisiran	Onpattro®	Alnylam	2018	GUGUGUUGGUGUUUGGUAC (21 mer)	hATTR	TTR 3'UTR	LNP
		Givosiran	Givlaari®	Alnylam	2019	UGCUUUAGUAUGUUUGCUA (21 mer)	AHP	ALAS1 mRNA exon 5	GalNAc
		Inclisiran	Leqvio®	Novartis	2020	UGCUGUACUUGCCGCACAG (21 mer)	HeFH	PCSK9 mRNA exon 1	GalNAc
siRNA	RISC via Ago2	Lumasiran	Oxlumo®	Alnylam	2020	AGGUUGGCUCUACUUGCUA (21 mer)	PH1	HAO1 mRNA exon 5	GalNAc
		Vutrisiran ^a^	Amvuttra®	Alnylam	2022	GUGUGUUGGUGUUUGGUAC (21 mer)	hATTR	TTR 3'UTR	GalNAc
		Nedosiran	Rivfloza®	Novo Nordisk	2023	CUCUUUACAAACACGACUA (21 mer)	PH1	LDHA mRNA exon 5	GalNAc
		Fitusiran	Qfitlia®	Sanofi	2025	GUGUGUUGGUGUUUGGUAC (21 mer)	Hemophilia A or B	SERPINC1 exon 2	GalNAc
	Exon inclusion	Nusinersen	Spinraza®	Ionis Pharma, Biogen	2016	UCACUUUCAUAAUGCUGG (18 mer)	SMA	SMN2 pre-mRNA exon 7	Nacked
		Eteplirsen	Exondys 51®	Sarepta Tx	2016	CUCCAACAUCAAGGAAGAUGGCAUUUCUAG (30 mer)	DMD	DMD pre-mRNA exon 51	Nacked
		Milasen	-	Boston Children’s Hospital	2018	AATGTTAGTGCTTGTTGAGGGC (22 mer)	CLN7	MFSD8 pre-mRNA exon 5	Nacked
	Exon skipping	Golodirsen	Vyondys 53®	Sarepta Tx	2019	GUUGCCUCCGGUUCUGAAGGUGUUC (25 mer)	DMD	DMD pre-mRNA exon 53	Nacked
		Viltolarsen	Viltepso®	Nippon Shinyaku Pharma	2020	CCUCCGGUUCUGAAGGUGUUC (21 mer)	DMD	DMD pre-mRNA exon 53	Nacked
		Casimersen	Amondys 45®	Sarepta	2021	CAAUGCCAUCCUGGAGUUCCUG (22 mer)	DMD	DMD pre-mRNA exon 45	Nacked
ASO		Formivirsen ^b^	Vitravene®	Ionis Pharma, Novartis	1998	GCGTTTGCTCTTCTTCTTGCG (21 mer)	CMV retinitis with AIDS	CMV IE-2 mRNA	Nacked
		Mipomersen ^b^	Kynamro®	Ionis Pharma, Genzyme, Kastle Tx	2013	GCCUCAGTCTGCTTCGCACC (20 mer)	HoFH	Apolipoprotein B-100 mRNA exon 26	Nacked
		Inotersen	Tegsedi®	Ionis Pharma, Akcea Pharma	2018	UCUUGGTTACATGAAAUCCC (20 mer)	hATTR	TTR 3'UTR	Nacked
	RNase H1-mediatedmRNA degradation	Volanesorsen	Waylivra®	Ionis Pharma, Akcea Pharma	2019	AGCUUCTTGTCCAGCUUUAU (20 mer)	FCS	APOCIII mRNA exon 22	Nacked
		Eplontersen	Wainua®	AstraZeneca, Ionis Pharma	2023	UCUUGGTTACATGAAAUCCC (20 mer)	hATTR	TTR 3'UTR	GalNAc
		Tofersen	Qalsody®	Ionis Pharma, Biogen	2023	CAGGATACATTTCTACAGC (20 mer)	ALS	SOD1 mRNA exon 2	Nacked
		Olezarsen	Tryngolza®	Inois Pharma	2024	AGCUUCTTGTCCAGCUUUAU (20 mer)	FSC	APOC3 mRNA exon 2	GalNAc
	Telomerase inhibition	Imetelstat	Rytelo®	Geron Corporation	2024	TAGGGTTAGACAA (13 mer)	MDS	hTERC	Nacked
	VEGF inhibition	Pegaptanib ^b^	Macugen®	Pfizer, Eyetech	2004	CGGAAUCAGUGAAUGCUUAUACAUCCG (28 mer)	wAMD	VEGF165 protein	PEG
Aptamer	Endothelial protection	Defibrotide sodium	Defitelio®	Jazz Pharma	2016	GGTTGGATTGGTTGG and GGTTGGATCGGTTGG (9-80 mer)	VOD	Thrombin	PEG
	Complement C5 inhibition	Avacincaptad pegol	IZERVAY®	Archemix, Iveric Bio	2023	CGCCGCGGUCUCAGGCGCUGAGUCUGAGUUUACCUGCG (36 mer)	GA	C5 protein	PEG

^a^ ALN-TTRSC02, alternative name for Vutrisiran.

^b^ withdrawn upon FDA approval.

AHP, Acute Hepatic Porphyria; AIDS, Acquired immune deficiency sysndrome; ALS, Amyotrophic Lateral Sclerosis; ASO, antisense oligonucleotide; CLN7, Neuronal Ceroid Lipofuscinosis 7; CMV, Cytomegalovirus; DMD, Duchenne Muscular Dystrophy; FCS, Familiar Chylomicronemia Syndrome; GA, Geographic Atrophy; GalNAc, N-acetylgalactosamine; hATTR, Hereditary Transferin-mediated amyloidosis; HeFH, Heterozygous Familiar Hypercholesterolemia; HoFH, Homozygous Familiar Hypercholesterolemia; MDS, Myelodysplastic Syndromes; PEG, Polyethylene Glycol; PH1, Primary Hyperoxaluria type 1; RISC, RNA-induced silencing complex; SMA, Spinal Muscular Atrophy; VOD, Veno-occlusive disease; wAMD, Wet Age-Related Macular Degeneration.

## 2. RNA processing and the journey from RNA to protein

**Figure 2.**
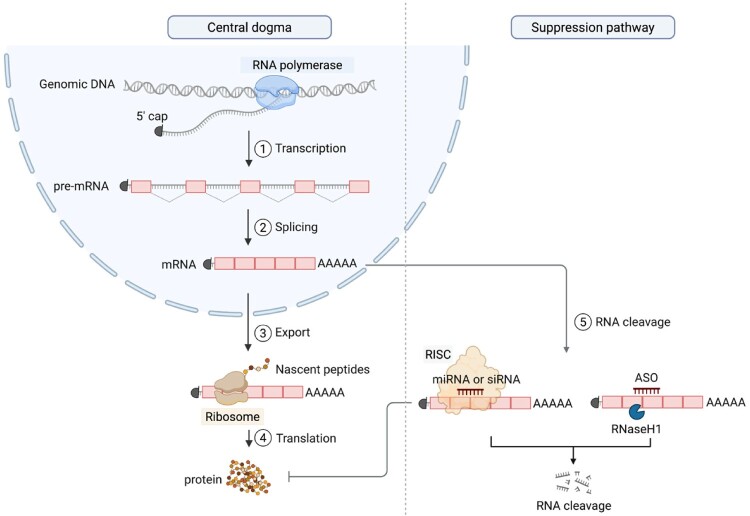
Mechanistic overview of RNA processing from DNA transcription to protein synthesis. A schematic of the central dogma and RNA silencing pathways in eukaryotic cells. On the left, RNA polymerase II transcribes genomic DNA into pre-mRNA, which undergoes 5′ capping, splicing to remove introns (steps 1 and 2), and polyadenylation to form mature RNA (mRNA). The processed mRNA is then exported to the cytoplasm (step 3), where it is translated into protein by ribosomes (step 4). On the right, RNA silencing pathways are depicted: mRNA is cleaved by RNA-induced silencing complex (RISC) guided by micro RNA (miRNAs) or small interfering RNAs (siRNAs), or by RNase H following antisense oligonucleotides (ASOs) binding (step 5). These mechanisms reduce mRNA abundance and inhibit protein synthesis through post-transcriptional regulation.

RNA processing encompasses multiple steps—transcription, capping, splicing, polyadenylation, export, and RNA cleavage—all of which are tightly regulated to ensure correct gene expression (Figure 2). Among these, pre-mRNA splicing plays a critical role not only in ensuring a diversity of transcripts but also in RNA fate through nuclear export, stability, and adjustment of translation efficiency (Shenasa and Bentley (2023), Reimer et al. (2021)). Increasing evidence has linked RNA isoforms to disease pathogenesis, driving efforts to identify disease-associated RNA isoforms across various disorders.

In eukaryotic cells, RNA polymerase II transcribes DNA into premature mRNA (pre-mRNA), which undergoes processing steps such as 5′-capping, splicing, and polyadenylation. The mature mRNA is then exported to the cytoplasm for translation by ribosomes. The abundance of mRNA is also controlled by cleavage mechanisms, including RNase H- and RISC-mediated degradation, enabling dynamic regulation based on cell type and environmental context. Disruption of mRNA processing mechanisms, such as aberrant splicing or defective decay, can lead to the production of nonfunctional or truncated proteins and is frequently linked to disease.

Recent advances in RNA biology and analytical techniques have driven the development of RNA-based therapeutics, including siRNA, ASOs, splicing-switching oligonucleotides (SSOs), and RNA aptamers. In this review, we briefly introduce RNA biology, including RNA cleavage mechanisms relevant to ASOs and siRNAs, RNA splicing central to SSOs, and the structural basis of RNA aptamers, to provide a solid foundation for understanding these therapeutic strategies.

## 3. RNA cleavage through endogenous nucleases

### 3.1. Gene silencing mechanisms of antisense oligonucleotides and small interfering RNAs

**Figure 3.**
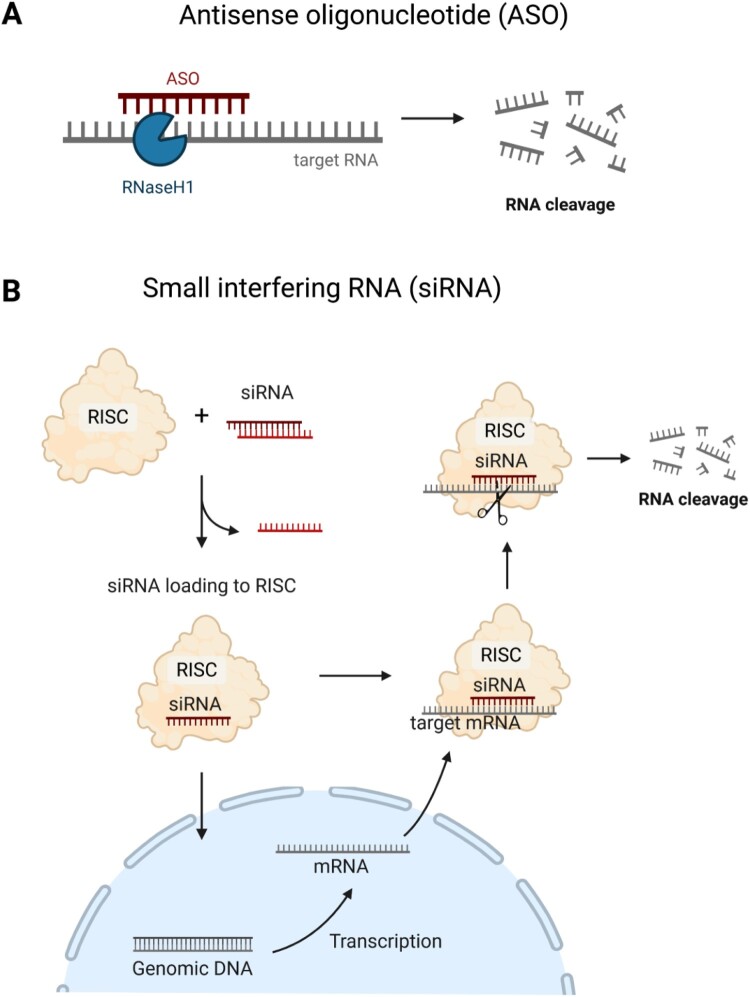
Gene silencing mechanisms of antisense oligonucleotides and small interfering RNAs. (A) Antisense oligonucleotides (ASOs) bind to complementary sequences on target RNAs, forming an RNA/DNA duplex. This duplex is recognized by RNase H, which cleaves the RNA strand, leading to degradation of the target transcript. (B) Small interfering RNAs (siRNAs) are processed to mature single-stranded siRNAs. Then, siRNAs are incorporated into the RNA-induced silencing complex (RISC). The siRNA–RISC complex binds to target mRNA sequences, leading to endonucleolytic cleavage, thereby suppressing gene expression post-transcriptionally.

RNase H-mediated RNA degradation is a post-transcriptional regulatory mechanism that controls gene expression (Figure 3A). RNase H is an endonuclease that specifically cleaves the RNA strand of RNA/DNA hybrids, thereby reducing the abundance of target transcripts. This mechanism underlies the activity of ASOs, which are synthetic single-stranded DNA molecules designed to hybridize with complementary RNA and induce its degradation. ASOs can function in both the nucleus and the cytoplasm and tolerate certain mismatches in sequence, allowing the modulation of a broad range of transcripts. Several ASO-based drugs, such as inotersen and eplontersen, have received regulatory approval for treating both genetic diseases and acquired diseases like lipid metabolism disorders (Kim and Krainer (2023)).

Another post-transcriptional mechanism is RNA interference (RNAi), a conserved gene silencing process involved in various biological functions such as tumorigenesis, inflammation, and defense against pathogens, including viruses (Shabalina and Koonin (2008)). Small interfering RNAs (siRNAs) are a representative example to silence disease-related genes in a sequence-specific manner (Figure 3B) (Hu et al. (2020)). Immature double-stranded RNAs are processed by RNase III enzymes (Dicer and Drosha) into siRNA duplexes, which are loaded into the RNA-induced silencing complex (RISC). The guide strand directs RISC to complementary RNAs, leading to cleavage by RNase H-like endonuclease Argonaute 2 (AGO2) (Santhekadur and Kumar (2020), Nakanishi (2024)). Such cleavage requires perfect or near-perfect base-pairing between the siRNA and its target, ensuring high specificity.

ASOs and siRNAs offer complementary strategies for therapeutic gene silencing. ASOs act in an RISC-independent manner and tolerate mismatches, making them suitable for targeting nuclear transcripts or modulating splicing (Collotta et al. (2023)). In contrast, siRNAs act in the cytoplasm through RISC and provide potent, highly specific silencing at low doses. Chemical modifications that enhance stability, specificity, and delivery have expanded the therapeutic potential of both RNA-based platforms. Consequently, ASO- and siRNA-based drugs now play a central role in RNA therapeutics, with ongoing clinical trials and regulatory approvals confirming their efficacy and safety for treating human diseases.

### 3.2. FDA-approved antisense oligonucleotide therapeutics

ASOs have been increasingly applied to the treatment of various diseases, such as genetic, neurological, and cardiovascular disorders (Kim and Krainer (2023), Lauffer et al. (2024), Bajaj and Rader (2020)). Advances in chemical modifications and delivery systems have expanded their therapeutic potential (Egli and Manoharan (2023)). First-generation ASOs, such as fomivirsen, faced challenges such as rapid degradation by endogenous nucleases. To address this, chemical modifications were introduced into second-generation ASOs, including 2′-O-methoxyethyl (MOE) groups and phosphorothioate backbone linkages, enhancing their nuclease resistance and stability. Further advances have led to the development of third-generation ASOs, incorporating novel chemical structures such as locked nucleic acids, peptide nucleic acids, and morpholino phosphoramidate, along with bio-conjugations and nanocarrier formulations to improve the affinity of hybridization to target RNA, pharmacokinetic profiles, and therapeutic efficacy. However, no third-generation ASO has yet received FDA approval.

While ASO therapeutics initially developed for central nervous system disorders, including spinal muscular atrophy (SMA) (Rinaldi and Wood (2018)), advances in conjugate technologies such as N-acetylgalactosamine (GalNAc) ligands have significantly broadened their range of applications to systemic diseases by enabling targeted delivery to hepatocytes (Collotta et al. (2023)). ASOs offer key advantages, including high sequence specificity, adjustable design to address diverse genetic mutations, and the ability to target previously undruggable RNAs (Lauffer et al. (2024)). These features, combined with improved stability and delivery systems, underscore the expanding therapeutic versatility of ASOs. In this context, we investigated FDA-approved ASO drugs, their molecular targets, associated diseases, and clinical efficacy.

#### 3.2.1. Fomivirsen (Vitravene®)

Fomivirsen (Vitravene®) was the first ASO drug to be approved by the FDA (in 1998). It was developed to treat cytomegalovirus (CMV) retinitis in patients with acquired immunodeficiency syndrome (AIDS). CMV retinitis is a severe retinal infection that can lead to blindness, particularly in the immunocompromised (Study G (2002)). Although the FDA has withdrawn its approval of fomivirsen due to declining demand resulting from the widespread use of highly active antiretroviral therapy (HAART) (Piper et al. (2000)), the field of antisense therapeutics has continued to evolve, experiencing periods of both progress and setbacks.

#### 3.2.2. Mipomersen (Kynamro®)

Mipomersen (Kynamro®) is a second-generation ASO composed of 20 nt (Bell et al. (2011), Hair et al. (2013)). It specifically targets the mRNA of apolipoprotein B100 (ApoB-100), an essential structural component of atherogenic lipoproteins. Very-low-density lipoprotein (VLDL) and low-density lipoprotein (LDL) function as ligands for LDL receptor-mediated endocytosis (Raal and Santos (2012)). Mipomersen is predominantly distributed to the liver, where it inhibits apoB-100 expression in hepatocytes. This in turn reduces VLDL production and, consequently, lowers the plasma levels of LDL cholesterol and ApoB-containing lipoproteins (Bell et al. (2011)). Mipomersen was approved by the FDA for the treatment of homozygous familial hypercholesterolemia (HoFH), a rare genetic disorder characterized by markedly elevated LDL-C levels and a significantly increased risk of premature atherosclerotic cardiovascular disease (ASCVD) (Toth (2013)). Despite its efficacy in lowering LDL-C, mipomersen was withdrawn from the US market due to safety concerns. Since mipomersen, ASOs have emerged as promising therapeutics for treating liver diseases such as NAFLD, hepatitis, fibrosis, and liver cancer, owing to their high efficiency, low toxicity, and effectiveness at low dosages (Lu et al. (2022)).

#### 3.2.3. Inotersen (Tegsedi®) and eplontersen (Wainua®)

Inotersen, approved by the FDA as an orphan drug in 2018, is an ASO that inhibits the synthesis of transthyretin (TTR) protein by binding to a specific RNA sequence. Meanwhile, eplontersen, developed with the same target and mechanism of action, is a second-generation ASO conjugated with a triantennary GalNAc, enabling selective hepatocyte uptake via the asialoglycoprotein receptor (ASGPR) (Brannagan et al. (2022)). The targeted delivery confers 30- to 50-fold greater potency in mice compared with that of inotersen, despite allowing lower and less frequent dosing (Viney et al. (2021)). Inotersen requires more intensive monitoring because of risks such as thrombocytopenia (low platelet count) and potentially life-threatening glomerulonephritis (Dyck et al. (2020)). Eplontersen has a more favorable safety profile, although vitamin A supplementation is required to address the associated reductions in serum retinol levels.

#### 3.2.4. Volanesorsen (Waylivra®) and olezarsen (Tryngolza®)

Volanesorsen (Waylivra®) is a second-generation 2′-O-methoxyethyl (MOE) chimeric ASO that was approved by the FDA in 2019 (Paik and Duggan (2019)). It is indicated for treating familial chylomicronemia syndrome (FCS), a rare inherited disorder often caused by mutations in the *lipoprotein lipase (LPL*) gene. It targets *apolipoprotein C3 (APOC3)* mRNA, thereby reducing the expression of the protein it encodes. APOC3 protein is a key regulator of lipid metabolism, as it inhibits LDL activity and the hepatic uptake of triglyceride-rich lipoprotein, ultimately impairing lipolysis (Ooi et al. (2008)). By lowering APOC3 levels, volanesorsen restores LPL activity and significantly reduces serum triglyceride concentration.

Olezarsen (Tryngolza®) is a next-generation ASO designed to target the same *APOC3* mRNA sequence as volanesorsen but incorporating a GalNAc conjugate to enhance liver-specific delivery. This targeted approach enables the selective inhibition of APOC3 synthesis in hepatocytes at lower doses, improving therapeutic efficacy while minimizing systemic side effects such as thrombocytopenia (Alexander et al. (2019), Tardif et al. (2022)). Clinical studies have demonstrated that olezarsen effectively lowers APOC3 levels and serum triglycerides, particularly in patients with cardiovascular disease or at high risk of it (Stroes et al. (2024)). Together, volanesorsen and olezarsen exemplify the advance of ASO therapies associated with lipid metabolism, reflecting a shift from systemic action to targeted, liver-specific delivery that enhances potency and safety.

#### 3.2.5. Tofersen (Qalsody®)

Tofersen, marketed as Qalsody®, is a copper–zinc superoxide dismutase 1 (SOD1) mRNA-targeting ASO drug that reduces the production of misfolded SOD1 protein (Jin and Zhong (2023)). It was approved by the FDA in 2023 for the treatment of amyotrophic lateral sclerosis (ALS) associated with mutations in the *SOD1* gene. *SOD1* encodes a 16-kDa protein that functions as a homodimer, playing multiple roles in cellular homeostasis. In addition to its enzymatic function in detoxifying superoxide radicals, SOD1 acts as a transcription factor under oxidative stress (Xu et al. (2022), Eleutherio et al. (2021)). Pathogenic variants in the *SOD1* gene, first linked to ALS in 1993, disrupt the proper folding, metal binding, and stability of the SOD1 protein. These alterations result in misfolded protein aggregates that exert toxic gain-of-function effects through mechanisms such as proteasome inhibition, mitochondrial dysfunction, and ER stress (Benatar et al. (2025); Berdynski et al. (2022); Huai and Zhang (2019)). Over 150 *SOD1* mutants have been identified (Abel et al. (2012)), and misfolded SOD1 protein has been consistently detected in degenerating motor neurons and glial cells in ALS patients and animal models (Opie-Martin et al. (2022)). Some mutations can trigger the production of truncated proteins that escape nonsense-mediated mRNA decay (Guissart et al. (2020)). Tofersen specifically targets *SOD1* mRNA variants, including the common European *SOD1* variant p.D91A (c.272A > C). In a recent study, five out of six patients with this mutation showed reduced levels of serum neurofilament light chain—a biomarker of neuronal injury—and exhibited slower ALS progression after being treated with tofersen (Weishaupt et al. (2024)). These findings support tofersen as a promising therapeutic approach for a genetically defined subset of ALS patients.

### 3.3. FDA-approved small interfering RNA therapeutics

Small interfering RNAs (siRNAs) are double-stranded RNA molecules that mediate gene silencing through the RNA interference (RNAi) pathway. This pathway is highly conserved across eukaryotes to ensure effective defense against viral RNAs. Upon entering the cytoplasm, siRNA is incorporated into the RNA-induced silencing complex (RISC), where the guide strand directs the complex to complementary mRNA targets (Figure 2). The key catalytic component AGO2 leads to the cleavage of target mRNA and subsequent gene silencing. This mechanism allows siRNAs to achieve highly specific and efficient inhibition of disease-related genes, making them attractive candidates for therapeutic development. To enhance tissue targeting and reduce off-target effects, all FDA-approved siRNA therapeutics have been engineered to be conjugated to GalNAc for targeted delivery to hepatocytes.

#### 3.3.1. Patisiran (Onpattro®) and vutrisiran (Amvuttra®)

Patisiran was the first siRNA to be approved by the FDA (in 2018) for the treatment of hereditary transthyretin-mediated amyloidosis (hATTR) (Hoy (2018)). hATTR is a progressive and multisystemic disorder characterized by the deposition of misfolded transthyretin (TTR) amyloid fibrils in the peripheral nervous system, heart, and other organs (Poli et al. (2023)). Patisiran was formulated in lipid nanoparticles for hepatocyte-specific delivery to reduce *TTR* mRNA (Suzuki and Ishihara (2021)). *TTR* mRNA is transcribed in the liver and translated into TTR protein, which misfolds when it contains pathogenic mutations. Patisiran utilizes a double-stranded siRNA complementary to *TTR* mRNA, leading to cleavage by the RISC and degradation of the target transcript. Patisiran significantly reduces circulating TTR levels and slows or reverses the progression of polyneuropathy in patients with hATTR. Vutrisiran shares the same TTR mRNA target and antisense sequence as patisiran, but uses advanced GalNAc-based delivery chemistry, allowing it to be injected subcutaneously and to be dosed at longer intervals (Keam (2022)). Vutrisiran was approved by the FDA in 2022 and demonstrated comparable efficacy to patisiran, with a more patient-friendly administration route and quarterly dosing.

#### 3.3.2. Givosiran (Givlaari®)

Givosiran targets aminolaevulinic acid synthase 1 (*ALAS1*) mRNA in hepatocytes, promoting its degradation via the RISC pathway. This process reduces ALAS1 protein levels and suppresses the accumulation of toxic intermediates. It was approved by the FDA in 2019 for the treatment of acute hepatic porphyria (AHP), a group of rare genetic disorders characterized by defects in heme biosynthesis (Scott (2020)). As conjugated to GalNAc, Givosiran achieves liver-specific delivery via ASGPR-mediated uptake without the use of lipid nanoparticles.

#### 3.3.3. Inclisiran (Leqvio®)

Inclisiran targets *proprotein convertase subtilisin/kexin type 9 (PCSK9)* mRNA to inhibit its expression in hepatocytes to lower LDL cholesterol (Warden and Duell (2021)). Elevated LDL cholesterol is a significant risk factor for atherosclerotic cardiovascular disease. PCSK9 promotes the degradation of LDL receptors (LDLRs), reducing hepatic LDL uptake. However, inclisiran provides a long-acting therapeutic option for LDL-C reduction, with dosing being performed only twice a year (Ray et al. (2023)). Inclisiran exemplifies the use of siRNA for chronic metabolic diseases and demonstrates durable pharmacodynamic effects with infrequent dosing schedules. It was approved for heterozygous familial hypercholesterolemia (HeFH) by the FDA in 2021 (Lamb (2021)).

#### 3.3.4. Lumasiran (Oxlumo®)

Lumasiran is an siRNA therapeutic that targets hydroxyacid oxidase 1 (*HAO1*) mRNA to reduce the expression of glycolate oxidase, an upstream enzyme in oxalate biosynthesis (Goga and Stoffel (2022)). It was approved by the FDA in 2020 for the treatment of primary hyperoxaluria type 1 (PH1), a rare autosomal recessive disorder characterized by excessive hepatic oxalate production due to a deficiency in alanine-glyoxylate aminotransferase (AGT) (Kukreja et al. (2019), Scott and Keam (2021)). In the absence of AGT, the activity of glycolate oxidase leads to oxalate overproduction. By silencing HAO1, lumasiran effectively decreases urinary oxalate levels. Lumasiran offers a disease-modifying approach that bypasses the defective AGT enzyme by inhibiting the upstream metabolic pathway.

#### 3.3.5. Nedosiran (Rivfloza®)

Nedosiran is an siRNA therapeutic that targets lactate dehydrogenase A (*LDHA*) mRNA to reduce hepatic oxalate production. LDHA catalyzes the conversion of glyoxylate to oxalate, and its inhibition offers a unified therapeutic strategy for primary hyperoxaluria (PH) types 1, 2, and 3 by bypassing upstream enzymatic defects (Goga and Stoffel (2022), Ariceta et al. (2021)). By silencing LDHA, Nedosiran diverts glyoxylate away from the oxalate-producing pathway, providing a genotype-independent approach to treatment of PH. It was approved by the FDA for PH1 and is under evaluation for broader application to PH2 and PH3 (Syed (2023)).

#### 3.3.6. Fitusiran (Qalsody®)

Fitusiran targets antithrombin (*SERPINC1*) mRNA and was developed for the prophylactic treatment of hemophilia A and B, including patients with inhibitors that neutralize the activity of replacement factor VIII or IX (Lu et al. (2025)). Hemophilia is characterized by a deficiency in clotting factor VIII (A) or IX (B), leading to impaired thrombin generation and prolonged bleeding. Elevated antithrombin levels further suppress clotting. Fitusiran reduces the expression of *SERPINC1* mRNA, lowering antithrombin levels and rebalancing hemostasis by enhancing thrombin generation. It was approved by the FDA in 2025 as a prophylactic treatment for hemophilia A or B in adults and adolescents aged 12 years or older (Lee (2025)).

## 4. Splicing regulation

### 4.1. Modulation of pre-mRNA splicing

Splicing is a fundamental step in eukaryotic gene expression that involves removing introns from pre-mRNA, immature RNA, to produce mature messenger RNA (mRNA). Splicing is orchestrated by the spliceosome complex with the assistance of ribonucleoproteins (RBPs) that either promote or repress splicing. The core spliceosome components consist of five small nuclear ribonucleoproteins (snRNPs)—U1, U2, U4, U5, and U6—which are uridine-rich and recognize conserved splice sites and a branch point, assembling in a stepwise manner to excise introns and ligate exons (Nguyen et al. (2015), Montemayor et al. (2014)).

Abnormal splicing is increasingly recognized as a major cause of various diseases, including cancer, muscular dystrophies, and neurodegenerative disorders. One common mechanism involves the production of nonfunctional or toxic splice isoforms due to mutations at critical splice site sequences or within spliceosome components. For example, mutations in the 3′ splice site (3′ss) or U2 snRNP can block the transition from spliceosome complex B to the catalytically active complex B*, resulting in exon skipping or intron retention (Nino et al. (2022)). Such splicing defects are implicated in pediatric neurological diseases like SMA and Duchenne muscular dystrophy (DMD), both of which involve misregulated exon inclusion.

Given the clinical relevance of splicing dysregulation, particularly at the 3′ss, there is growing interest in therapeutic strategies that correct splicing defects at the RNA level. Among these, SSOs have emerged as a promising approach. SSOs are similar to conventional ASOs but are designed to bind to pre-mRNA near aberrant splice sites or regulatory elements to correct abnormal splicing patterns (Havens and Hastings (2016)). By sterically blocking access to splicing silencers or enhancers, SSOs can restore correct exon inclusion or promote the skipping of disease-associated exons without requiring RNase-mediated RNA degradation (Collotta et al. (2023), Havens and Hastings (2016)). This mechanism allows the restoration of functional protein expression or suppression of deleterious isoforms, offering a targeted and reversible therapeutic strategy. We review the therapeutic applications of FDA-approved SSOs in diseases caused by aberrant splicing.

### 4.2. FDA-approved splice-switching oligonucleotide therapeutics

SSOs represent a novel class of RNA-based therapeutics that modulate pre-mRNA splicing to correct or bypass genetic defects. Nusinersen, the first FDA-approved SSO, established the clinical feasibility of correcting aberrant splicing to treat SMA at the RNA level. Since then, additional SSOs such as eteplirsen, golodirsen, and viltolarsen have been approved for Duchenne muscular dystrophy (DMD), while casimersen targets a different exon in the same disease. These therapeutics work by restoring the production of functional proteins or by suppressing the expression of toxic protein variants that contribute to disease pathology. The clinical success of nusinersen for SMA and eteplirsen for DMD highlights the therapeutic potential of this approach. As we deepen our understanding of splicing regulation alongside ongoing progress in delivery technologies, SSOs are on the cusp of expansion into a broader range of disease applications. These advances have established SSOs as a rapidly evolving and transformative platform in precision medicine.

#### 4.2.1. Nusinersen (Spinraza®)

**Figure 4.**
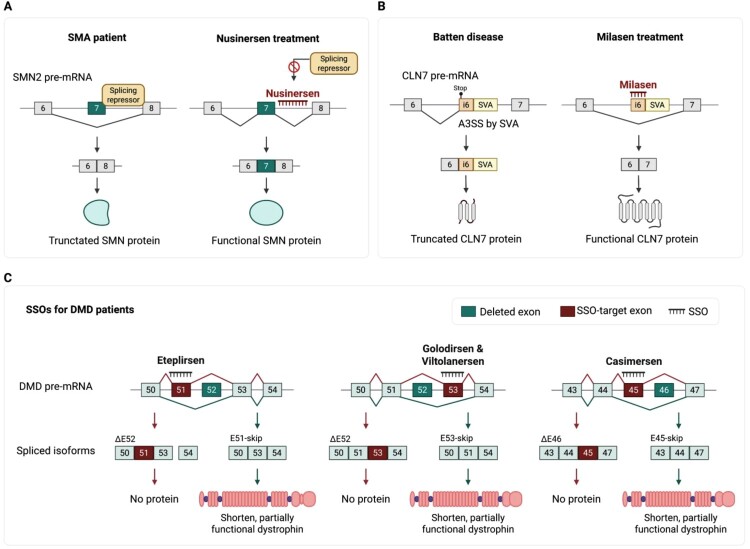
Therapeutic applications of splice-switching oligonucleotides. (A) Mode of action of nusinersen, which was approved by the FDA in 2016. In patients with spinal muscular atrophy (SMA), exon 7 exclusion of *SMN2* results in the production of a truncated and nonfunctional SMN protein. Nusinersen promotes exon inclusion by blocking the binding of the splicing repressor at intron 7, thereby restoring functional SMN protein production. (B) Mode of action of milasen, which was approved by the FDA in 2018. In Batten disease, a short interspersed nuclear element (SINE)-variable number tandem repeat (VNTR)-Alu element, abbreviated as SVA, transposon insertion in intron 6 of the CLN7 gene disrupts splicing by causing misrecognition of the splice site, leading to truncated protein production. Milasen masks the aberrant splice site, restoring normal splicing and enabling functional CLN7 protein production. (C) FDA-approved SSOs for treating Duchenne muscular dystrophy (DMD), including eteplirsen, golodirsen, viltolarsen, and casimersen. Each SSO drug induces exon skipping to restore the reading frame of DMD transcripts, resulting in a shortened but functional dystrophin protein. Each drug targets different exons for skipping.

Nusinersen was the first SSO to be approved by the FDA (in 2016) and remains a widely used treatment for SMA. It promotes the inclusion of exon 7 in *SMN2* pre-mRNA to increase functional SMN protein production (Figure 4A) (Qiu et al. (2022)). SMA is an autosomal recessive disorder characterized by the progressive loss of motor neurons. It is classified into stages I–IV in terms of its severity (Kolb and Kissel (2015)). The disease is caused by homozygous loss of the *SMN1* gene. Although *SMN2* is nearly identical to *SMN1*, a single-nucleotide difference in exon 7 causes inefficient exon inclusion, leading to a nonfunctional truncated protein (Farrar et al. (2017)). The SMN protein is essential for U-rich snRNP assembly and axonal mRNA transport, particularly in motor neurons (Tisdale and Pellizzoni (2015)). Patients with milder forms of SMA often have more copies of *SMN2*, allowing greater SMN protein expression (Tisdale and Pellizzoni (2015), Wirth (2021)). Nusinersen corrects the splicing defect by binding to intron 7 of *SMN2* and blocking the recruitment of splicing repressors such as hnRNPA1/A2 (Hua et al. (2008)). The blocking promotes exon 7 inclusion, restores the correct distribution of polyadenylated RNA, and prevents structural abnormalities in motor neurons (Berciano et al. (2020)). Following the success of nusinersen, SSO platforms have been expanded beyond neurological disorders to other conditions such as cancers and metabolic diseases, highlighting their potential to correct splicing defects across a broad range of diseases (Kim and Krainer (2023), Rinaldi and Wood (2018), Xiong et al. (2021)).

#### 4.2.2. Milasen

Neuronal ceroid lipofuscinoses (NCLs), also known as Batten disease, are autosomal recessive neurodegenerative disorders caused by mutations in genes involved in lysosomal function (Carcel-Trullols et al. (2015)). Among these, mutations in CLN7 *(MFSD8)* lead to a late-infantile form of NCL, with symptoms typically appearing between the ages of 2 and 4 years (Carcel-Trullols et al. (2015); Kousi et al. (2012); Pasquetti et al. (2023)). CLN7 encodes a lysosomal chloride channel that regulates luminal pH and calcium release through interaction with the ion channel TRPML1 (Wang et al. (2021)). A notable personalized RNA therapeutic is Milasen, developed for a young patient named Mila with Batten disease. Whole-genome sequencing uncovered a novel intronic mutation in CLN7 caused by the insertion of foreign DNA, which activated a cryptic splice site, leading to intron 6 retention and loss of the functional protein (Kim et al. (2019)). Milasen, a 22-nt SSO, blocks the aberrant splice site and restores normal exons 6-exon 7 joining (Figure 4B). It was reported that, in patient-derived fibroblasts, Milasen corrected CLN7 splicing, reduced lysosomal vacuolization and mass, and improved autophagic flux (Kim et al. (2019)). Although no treatments have yet been approved for Batten disease, the Milasen case underscores the potential of personalized SSOs in treating rare splicing-driven disorders (Brudvig and Weimer (2022)), especially those involving noncoding or cryptic mutations that cannot be detected by conventional diagnostics.

#### 4.2.3. Phosphorodiamidate morpholino oligomer-based exon skipping for duchenne muscular dystrophy

DMD is a severe X-linked recessive neuromuscular disorder caused by mutations in the *DMD* gene. These mutations typically disrupt the translational reading frame or introduce PTCs, leading to the complete absence of the functional dystrophin protein (Gao and McNally (2015)). Dystrophin plays an essential role in linking cytoskeletal F-actin to the extracellular matrix via the dystrophin-associated glycoprotein complex (DAGC) (Gao and McNally (2015), Duan et al. (2021)). This connection helps maintain the structural integrity of muscle fibers throughout the mechanical stress of contraction (Gao and McNally (2015)). Dystrophin deficiency causes progressive muscular degeneration, with clinical symptoms appearing at around 2–3 years of age, followed by loss of ambulation and, ultimately, premature death due to cardiac or respiratory failure between the ages of 20 and 40.

Among the various *DMD* gene mutations, deletions involving exon 51 are the most common, accounting for approximately 13% of cases, followed by mutations in exon 45 (8.1%), exon 53 (7.7%), and exon 44 (6.2%) (Aartsma-Rus et al. (2009)). These mutations often cluster in specific hotspots, making exon-skipping therapy a viable strategy for restoring the in-frame status of transcripts and functional dystrophin protein production. The development and regulatory approval of SSOs designed to induce exon skipping represents a significant advance in personalized medicine for DMD.

#### 4.2.4. Eteplirsen (Exondys 51®)

Eteplirsen is the first exon-skipping therapeutic for DMD to be approved by the FDA in 2016, inducing exon 51 skipping in *DMD* pre-mRNA (Duan et al. (2021), Wilton-Clark and Yokota (2023)). It is composed of a phosphorodiamidate morpholino oligomer (PMO) backbone, a class of charge-neutral oligonucleotides that confer enhanced chemical stability and low toxicity (McDonald et al. (2021)). Eteplirsen restores the open reading frame in patients with deletions ending at exon 50 or starting at exon 52, representing approximately 14% of all DMD cases (Wilton-Clark and Yokota (2023)). Finally, eteplirsen enables the translation of a functional dystrophin protein with the internal deletion of exon 51 (Figure 4C). Despite its favorable safety profile, eteplirsen undergoes rapid systemic clearance and shows limited uptake into muscle, prompting efforts to improve tissue delivery and therapeutic efficiency (Lim et al. (2017), Akpulat et al. (2018)).

#### 4.2.5. Golodirsen (Vyondys 53®) and viltolarsen (Viltepaso®)

Subsequent PMO-based drugs targeting additional exons were developed to expand the number of treatable *DMD* mutations. Golodirsen received accelerated approval from the FDA in 2019 for patients amenable to exon 53 skipping (Figure 4C) (Aartsma-Rus and Corey (2020)). Golodirsen, compared to eteplirsen, has demonstrated greater restoration of dystrophin in patient muscle biopsies and improved localization of dystrophin to the sarcolemma, which contribute to slowing disease progression (Heo (2020), Frank et al. (2020)). Viltolarsen, a 21-mer PMO approved in 2020, binds to a distinct sequence within the same exon 53 region targeted by golodirsen and promotes the production of functional dystrophin protein (Figure 4C) (Aartsma-Rus and Corey (2020), Dhillon (2020)). Both drugs are applicable to patients with mutations that cause the skipping of exon 53, who collectively represent approximately 7.7% of DMD cases (deletion of exons 45–52, 47–52, 48–52, 49–52, 50–52, or exon 52 alone) (Watanabe et al. (2018)). Viltolarsen provides advantages in dose flexibility and cost-effectiveness because it is shorter than golodirsen.

#### 4.2.6. Casimersen (Amondys 45®)

Casimersen, the PMO that was most recently approved (in 2021), targets exon 45 of *DMD* pre-mRNA and is indicated for patients with mutations amenable to its skipping (Figure 4C) (Assefa et al. (2024), Shirley (2021)). Like other *DMD-*targeted SSOs, casimersen restores the reading frame, enabling the expression of an internally shortened but functional dystrophin protein (Assefa et al. (2024), Shirley (2021)). Phase III clinical trials for casimersen are currently underway in several countries worldwide.

Four FDA-approved therapies—eteplirsen, golodirsen, ciltolarsen, and casimersen—involve PMOs, which are neutrally charged molecules characterized by high stability and low toxicity, albeit with rapid clearance (McDonald et al. (2021)). Ongoing research has explored strategies to overcome this issue of rapid clearance, such as peptide-conjugated PMOs, chemically modified backbones, and nanoparticle-based delivery systems. In all of these cases, the aim has not been only to prolong the circulation time, but also to enhance tissue uptake and improve therapeutic efficacy.

## 5. RNA–protein interaction and regulatory roles of short RNAs

### 5.1. Aptamer technology for interaction with specific proteins

The term ‘aptamer’ is derived from the Latin *aptus* (to fit) and the Greek *meros* (particle), referring to their role as molecules of binding specifically to target ligands (Zhou and Rossi (2017)). Aptamers are short, single-stranded DNA or RNA oligonucleotides (typically 20–80 nt) that fold into distinct 3D structures, enabling them to bind specifically and with high affinity to target ligands (Ng et al. (2006), Mahmoudian et al. (2024)). These targets include intracellular proteins and peptides, small molecules (e.g. metal ions), pathogens (bacteria, viruses), and whole cells (Mahmoudian et al. (2024)). Unlike antibodies, which rely on sequence-based recognition, aptamers interact with targets via structural complementarity, allowing them to distinguish even subtle variations such as point mutations or structural differences caused by protein misfolding or conformational changes (Zhou and Rossi (2017)). They can also be chemically synthesized, offering scalable, cost-effective production with high batch consistency and enhanced stability during storage (Mahmoudian et al. (2024)).

**Figure 5.**
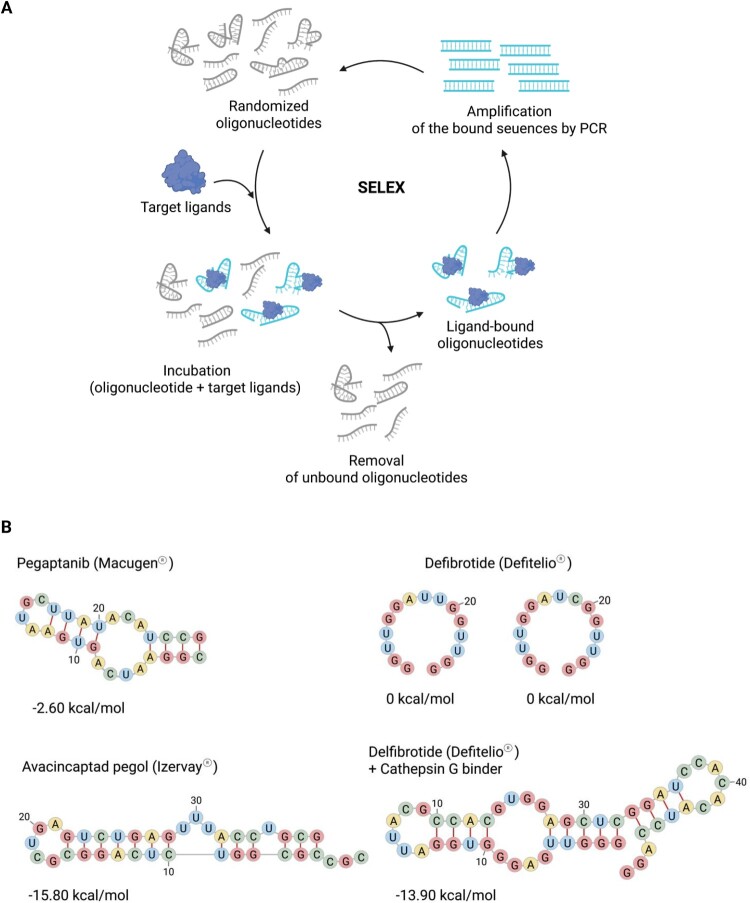
SELEX process for aptamer selection and secondary structures of FDA-approved RNA aptamers. (A) Schematic of the systematic evolution of ligands by the exponential enrichment (SELEX) process. A library of randomized oligonucleotides was incubated with target ligands, such as proteins, organic molecules, or cells. Ligand-bound oligonucleotides were separated from unbound sequences, and the bound sequences were amplified by PCR. This cycle was repeated to enrich the high-affinity aptamers. (B) Secondary structures of FDA-approved aptamers computationally predicted using RNAfold (ViennaRNA Package 2.0) (Lorenz et al. (2011)), with their minimum free energy (MFE) values shown below each structure. Nucleotides are color-coded, and short red lines indicate the complementary base pairs forming stems.

Aptamers are typically selected through the systematic evolution of ligands by exponential enrichment (SELEX) process, an in vitro iterative method that enriches high-affinity binders from an extensive oligonucleotide library (∼10^14^–10^15^ sequences) via repeated cycles of binding, separation, and amplification (Figure 5) (Zhou and Rossi (2017), Zhuo et al. (2017)). Recent advances have improved the *in vivo* stability, target specificity, and delivery systems of aptamers in general, thereby expanding aptamer applications beyond ocular disease to oncology, inflammation, and infectious diseases (Zhou and Rossi (2017), Zhuo et al. (2017), Buglak et al. (2020)). Aptamers have shown promise as precision therapeutics in cancer immunotherapy and radiotherapy by targeting the cell surface or acting as radiosensitizers (Gao et al. (2022)). Their development has also benefited from advances in high-resolution structural prediction and biomarker discovery (Mahmoudian et al. (2024), Zhuo et al. (2017), Ning et al. (2020), Cesarini et al. (2025)). With the progress in oligonucleotide chemistry and delivery platforms, aptamers are emerging as versatile therapeutics for both diagnostic and therapeutic uses (Jung et al. (2024)). Approaches such as aptamer–drug conjugates (AptDCs) and aptamer-functionalized nanoparticles aim to enhance the targeting precision and reduce off-target toxicity (Zhuo et al. (2017), Buglak et al. (2020), Cesarini et al. (2025)). The following section outlines mechanisms of FDA-approved aptamer drugs in aptamer-based therapies.

### 5.2. FDA-approved aptamer therapeutics

#### 5.2.1. Pegaptanib (Macugen®)

Pegaptanib is an RNA aptamer that specifically targets vascular endothelial growth factor A165 (VEGF165), a key isoform implicated in pathological angiogenesis (Ng et al. (2006)). It received FDA approval in 2004 for the treatment of neovascular (wet) age-related macular degeneration (Vinores (2006)). Pegaptanib specifically interacts with the heparin-binding domain unique to the VEGF165 isoform (Figure 5B) (Ng et al. (2006)). To enhance stability against nuclease degradation, it incorporates 2′-fluoro and 2′-O-methyl modifications on purine and pyrimidine bases (Ng et al. (2006)). Additionally, it is conjugated at the 5′ end to two branched 20-kDa polyethylene glycol (PEG) moieties, which prolongs the half-life and enhances bioavailability by reducing renal clearance (Vinores (2006)).

VEGF-A is a central regulator of angiogenesis and vascular permeability (Ferrara et al. (2003)). Alternative splicing of the *VEGF-A* gene generates multiple isoforms, among which VEGF165 is the predominant proangiogenic variant in disease states (Mamer et al. (2020), Harper and Bates (2008)). It binds to the tyrosine kinase receptors VEGFR1 and VEGFR2, promoting endothelial cell proliferation, migration, and neovascularization (Ferrara et al. (2003), Mamer et al. (2020)).

Clinically, isoform-selective targeting of pegaptanib allowed the treatment of wet AMD while potentially minimizing the effects on physiological angiogenesis mediated by the shorter forms (Gragoudas et al. (2004)). However, this specificity ultimately limited its efficacy because other VEGF-A isoforms also contribute to the disease. Specifically, compared with broader-spectrum anti-VEGF agents such as ranibizumab and aflibercept, pegaptanib showed inferior visual outcomes and reduced durability, leading to its eventual withdrawal from the market (Stein and Castanotto (2017)). Despite this, pegaptanib remains a fundamental example of aptamer-based drug design and clinical translation.

#### 5.2.2. Defibrotide (Defitelio®)

Defibrotide is a polydisperse mixture of single-stranded deoxyribonucleotides derived from porcine intestinal mucosa that binds thrombin and modulates coagulation pathways (Figure 5B) (Baker and Demaris (2016), Nauffal et al. (2022)). Further development of this agent incorporated cathepsin G-inhibiting aptamers with self-dimerizing capacity and TG tandem repeats to enhance stability and protease resistance (Figure 5B) (Gatto et al. (2008), Pescador et al. (2013)). Defibrotide received FDA approval in 2016 as the first and only treatment for hepatic veno-occlusive disease (VOD), a life-threatening complication following high-dose chemotherapy or hematopoietic stem cell transplantation (HSCT) (Corbacioglu et al. (2016), Richardson et al. (2017)). VOD develops in approximately 10%–15% of patients within 20–30 days and, without intervention, its mortality rate is exceptionally high, especially in cases with multi-organ failure (Richardson et al. (2017), Stein et al. (2016)).

Defibrotide inhibits thrombin-induced platelet aggregation and thromboxane formation in a manner dependent on its concentration (Baker and Demaris (2016), Bracht and Schror (1994)). It also modulates inflammatory and thrombotic signaling by interacting with adenosine receptors (A1, A2a, A2b), attenuating thrombin-induced endothelial dysfunction (Hassanian et al. (2014), Iyu et al. (2011)). In vitro studies using human umbilical vein endothelial cells have shown that defibrotide mitigates damage caused by chemotherapeutic agents (e.g. fludarabine) or serum starvation (Hassanian et al. (2014)). More recently, its endothelium-protective effects have sparked interest in applications beyond VOD, including vascular endotheliitis caused by infections such as SARS-CoV-2 (Calabretta et al. (2021), Hattori et al. (2022)). These emerging applications underscore the broader potential of defibrotide in both oncological and inflammatory vascular pathologies, warranting further clinical investigation (Pescador et al. (2013), Stein et al. (2016), Mitsiades et al. (2009)).

#### 5.2.3. Avacincaptad pegol (Izervay®)

Avacincaptad pegol is a PEGylated RNA aptamer that specifically targets complement protein C5, a key component of the terminal complement cascade during inflammation. The drug comprises a 40-kDa branched PEG moiety covalently attached to a 5′-end RNA sequence, enhancing stability and extending the intraocular half-life (Figure 5B). By binding to C5, avacincaptad pegol prevents proteolytic cleavage into C5a and C5b, thereby inhibiting the formation of the membrane attack complex (Danzig et al. (2024)). This blockade reduces inflammation and complement-induced cell lysis while preserving early complement activity (Ng and Powell (2021)). Because dysregulated complement activation contributes to the pathogenesis of AMD, particularly geographic atrophy (GA), C5 is a rational therapeutic target (Danzig et al. (2024)).

In a Phase 2/3 clinical trial, avacincaptad pegol reduced GA lesion progression by 14% over 12 months compared with the outcome of sham treatment, demonstrating both efficacy and safety (Khanani et al. (2023)). Based on these results, it was approved by the FDA for the treatment of GA secondary (Kang (2023)). The therapeutic potential of RNA aptamers in modulating complement pathways broadens the clinical application of oligonucleotide-based drugs beyond oncology and rare diseases.

## 6. Other RNA-based therapies

One notable RNA-based innovation is imetelstat (Rytelo®), the first FDA-approved telomerase inhibitor for hematological malignancies. Imetelstat is a 13-mer thio-phosphoramidate oligonucleotide covalently conjugated to a palmitoyl lipid, enhancing cellular uptake and nuclease resistance. Unlike conventional ASOs, imetelstat targets the RNA template component of telomerase (hTR/hTERC), blocking telomere elongation and inducing growth arrest in telomerase-dependent malignant cells (Jafri et al. (2016), Lennox et al. (2024)). In 2024, imetelstat received FDA approval for transfusion-dependent anemia in lower-risk myelodysplastic syndromes (MDSs), marking a milestone in RNA-targeted cancer therapeutics. It is also under investigation for high-risk myelofibrosis, showing promise in prolonging survival and reducing disease burden. Ongoing trials are exploring combinations with chemotherapeutics, and dosing regimens are also underway to mitigate dose-limiting hematological toxicities (Lennox et al. (2024)). Insights into telomerase biogenesis and trafficking may lead to optimized derivatives with improved safety and expanded indications.

## 7. Future directions in RNA biology for advancing RNA therapeutics

The rapid advancement of RNA-based therapeutics over the last decade has transformed molecular medicine. From ASOs and siRNAs to mRNA vaccines and RNA aptamers, RNA therapeutics have become versatile platforms with broad clinical applications. Their success in treating genetic diseases, cancer, viral infections, and rare disorders demonstrates their therapeutic potential to modify the disease process at a fundamental level. However, several key challenges are yet to be overcome to realize their full clinical potential.

One major challenge is improving stability and reducing immunogenicity through chemical modifications. Native nucleic acids are unstable in vivo and can trigger innate immune responses, leading to rapid degradation or systemic inflammatory reactions (Kong et al. (2023), Roh et al. (2025)). To overcome this, FDA-approved drugs such as PMOs use chemically modified backbones to resist nucleases and minimize immune activation (Egli and Manoharan (2023)). The study of RNA chemical modifications, or the epitranscriptome, which investigates naturally occurring chemical marks on RNA molecules, is rapidly evolving (Qiu et al. (2023); Kumar and Mohapatra (2021); Jeong et al. (2025)). Modifications such as N6-methyladenosine (m6A) and pseudouridine (ψ) modulate RNA stability, localization, and translation efficiency, offering new avenues for therapeutics (Boo and Kim (2020), Delaunay et al. (2024)). In parallel, novel RNA classes, including circular and enhancer RNAs, expand the functional complexity of the transcriptome and are being explored as therapeutic targets and delivery platforms (Chen et al. (2023); Li et al. (2023); O'Leary et al. (2025); He et al. (2021); Lee et al. (2020); Ju et al. (2023)). Their structural stability and unique regulatory properties not only deepen our understanding of RNA biology but also inspire designs for enhancing therapeutic efficacy.

Second, optimizing delivery systems is critical to ensuring cell-type specificity, reducing off-target effects, and enhancing intracellular uptake. GalNAc-conjugated siRNAs and ASOs effectively target the liver via the ASGPR (Paunovska et al. (2022)). This success has spurred the development of other bioconjugation strategies (Paunovska et al. (2022); Malecova et al. (2023); Wu et al. (2023)). Antibody-based delivery is advancing, focusing on smaller fragments like Fc or Fab to overcome size limitations (Jager et al. (2021)). Lipid conjugates and nanoparticle formulations have emerged as promising platforms for improving cellular uptake and tissue specificity (Mitchell et al. (2021)). These innovations support personalized RNA therapeutics guided by genomic and transcriptomic profiles.

Concurrently, RNA-based therapeutics have gained momentum not only for their sequence specificity and rapid development, but also for their diagnostic applications (Saju et al. (2025)). The COVID-19 pandemic underscored the value of RNA-based diagnostics for their speed, sensitivity, and cost-effectiveness, with advances in PCR, aptamer design, and CRISPR-based detection further strengthening their utility (Zhang et al. (2023), Sharma et al. (2021)). These advances point to a future where RNA-based systems enable disease monitoring, prediction, and precision intervention. Sequence-based strategies, informed by tissue-specific expression, spatial transcriptomics, and even extracellular RNA signatures, are increasingly viable.

In conclusion, RNA therapeutics have evolved from an experimental niche to a mainstream therapeutic class with growing clinical impact. As technological and biological advances converge, a new generation of highly specific, modular, and programmable RNA medicines is poised to address a wide range of diseases, including many previously untreatable. First-in-class agents exemplify this potential, laying the groundwork for safer, more potent, and personalized RNA-based interventions in the years ahead.
